# Ene–yne cross-metathesis with ruthenium carbene catalysts

**DOI:** 10.3762/bjoc.7.22

**Published:** 2011-02-04

**Authors:** Cédric Fischmeister, Christian Bruneau

**Affiliations:** 1 UMR 6226-CNRS-Université de Rennes 1, Sciences Chimiques de Rennes, Catalyse et Organométalliques, Campus de Beaulieu, 263 avenue du général Leclerc, 35042 Rennes cedex, France

**Keywords:** catalysis, cross-metathesis, enyne, fatty acid esters, ruthenium

## Abstract

Conjugated 1,3-dienes are important building blocks in organic and polymer chemistry. Enyne metathesis is a powerful catalytic reaction to access such structural domains. Recent advances and developments in ene–yne cross-metathesis (EYCM) leading to various compounds of interest and their intermediates, that can directly be transformed in tandem procedures, are reviewed in this article. In addition, the use of bio-resourced olefinic substrates is presented.

## Introduction

**Scheme 1:**
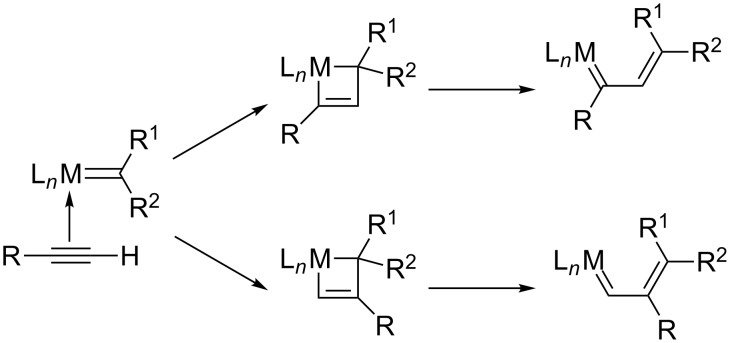
Interaction of triple bonds with a metal carbene.

The interaction of alkyne triple bonds with metal carbenes or metal vinylidene species was already known before the discovery of the very efficient molybdenum and ruthenium metathesis catalysts. In 1980, the polymerization of alkynes initiated by tungsten carbene was demonstrated by Katz [1–2] who proposed metallacyclobutenes as key intermediates in this polymerization. At the same period of time, Geoffroy [3] demonstrated that alkyne polymerization could be initiated directly from terminal alkynes without previous preparation of a metal carbene but via the formation of a reactive vinylidene tungsten species. Later on, the efficiency of ruthenium vinylidene precursors was also shown in olefin metathesis [4–10]. It is noteworthy that polymerization of terminal alkynes [11–13] and cyclotrimerization of triynes [14–20] with ruthenium carbene precursors is still a topic of current interest. Then, Fischer tungsten carbene complexes were used by Katz [21], and later Mori [22–23] utilized chromium alkoxycarbene to develop the first cyclizations via catalytic intramolecular enyne metathesis transformation. These initial works gave reason to postulate the interaction of metal carbene with alkyne to form a metallacyclobutene that rearranges to give a metal vinylcarbene (Scheme 1). This is the mechanistic basis of intramolecular enyne metathesis and EYCM reactions.

In this review, we will focus on recent developments in EYCM transformations with ruthenium carbene catalysts [24–32]. This will include some general features on EYCM. Examples involve ethylene, terminal olefins, cyclic olefins, diene metathesis with alkynes and finally applications in unsaturated fatty acid ester transformations.

## Review

### General considerations on EYCM

**Scheme 2:**
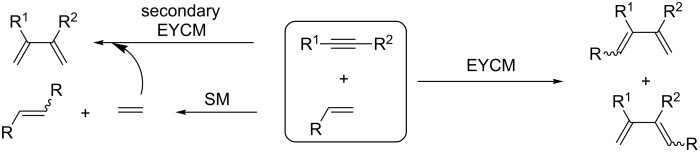
General scheme for EYCM and side reactions.

The EYCM is an attractive bimolecular transformation as it is an atom economical reaction which results in formal cleavage of a double bond and introduction of the two generated alkylidene fragments to the triple bond with formation of a conjugated 1,3-diene. However, this metathesis reaction is associated with some difficulties due to possible formation of several regio- and stereoisomers, as well as possible olefin self-metathesis (SM) and even secondary EYCM (Scheme 2). Though, at the moment the latter problems do not seriously appear to the best of our knowledge since the EYCM involving internal olefins has not been reported yet, except in the case of cyclic olefins.

**Figure 1:**
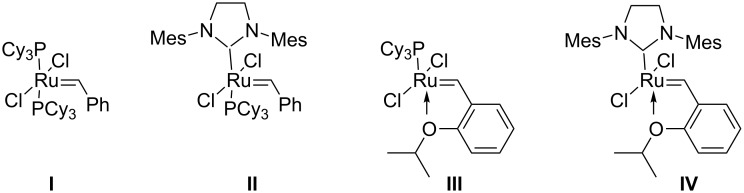
Selected ruthenium catalysts able to perform EYCM.

To date, most of the EYCM were performed using the first and second generation Grubbs (**I**, **II**) and Hoveyda (**III**, **IV**) catalysts (Figure 1).

**Scheme 3:**
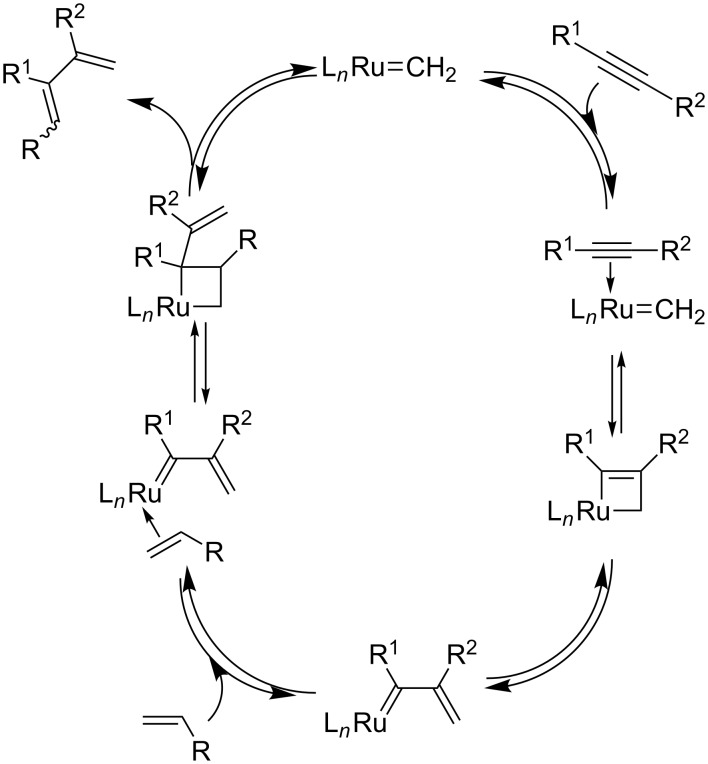
Catalytic cycle with initial interaction of a metal methylidene with the triple bond.

**Scheme 4:**
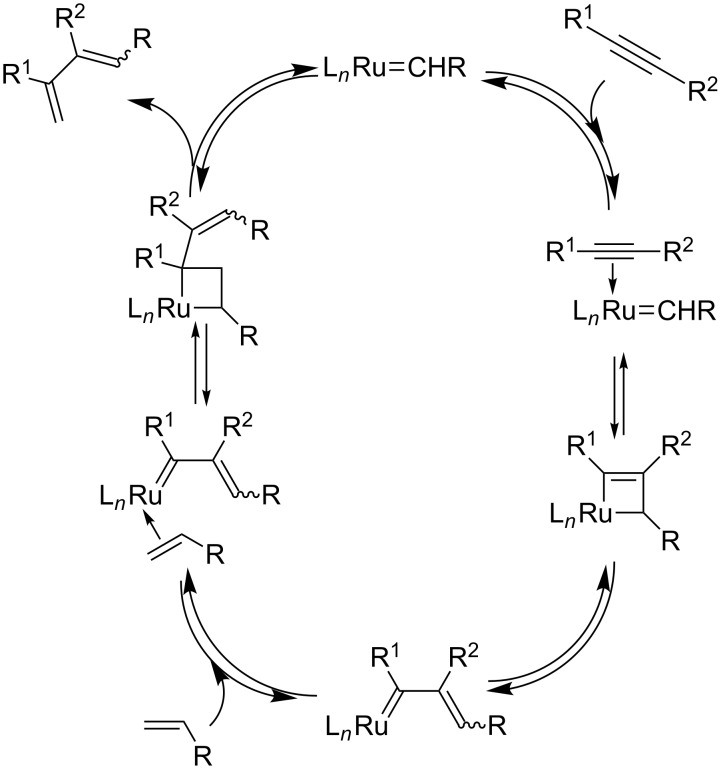
Catalytic cycle with initial interaction of a metal alkylidene with the triple bond.

Concerning the catalytic cycles, several pathways have been proposed. Mechanistic studies based on kinetic measurements assisted or not with calculations have been carried out for both the intra- and intermolecular ene–yne metathesis versions [33–38]. For EYCM the two pathways involve either an alkyne interaction with a methylidene metal species (Scheme 3) or an alkylidene metal intermediate (Scheme 4). In both cases, the ancillary ligands tricyclohexylphosphine or *N*-heterocyclic carbene have a crucial influence on the reaction, and the approach of the alkyne to the ruthenium center has to be controlled to obtain high regioselectivity. This corresponds to the exo/endo approaches reported in intramolecular ene–yne metathesis, which lead to cyclic products with different ring sizes.

EYCM with ruthenium catalysts was initiated in 1997 when Mori [39] and Blechert [40] reported the first examples with ethylene and higher olefins, respectively.

### EYCM with ethylene

**Scheme 5:**
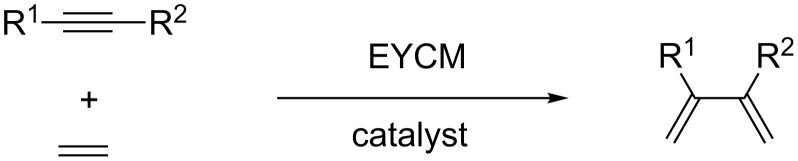
Formation of 2,3-disubstituted dienes via cross-metathesis of alkynes with ethylene.

The EYCM with ethylene is one of the simplest methods to generate conjugated dienes with two terminal methylene groups from alkynes (Scheme 5). In this special case there is no problem of regioselectivity and no risk of polluting the olefin formation as the self-metathesis of ethylene is non-productive. For these reasons, the reaction is highly selective.

**Figure 2:**
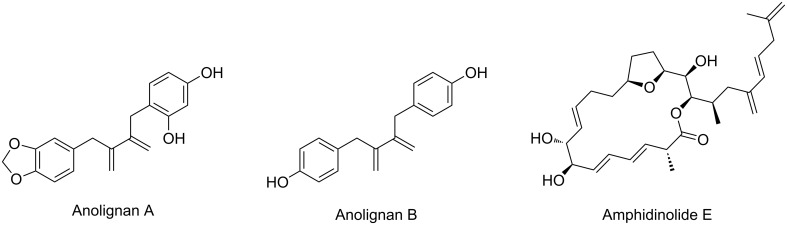
Applications of EYCM with ethylene in natural product synthesis.

The EYCM was initially performed with catalyst **I** under an atmosphere of ethylene at room temperature [39,41–42]. When the substrates were not reactive under these mild conditions, cross-metathesis efficiency was improved by using higher ethylene pressure [43] or changing the ruthenium precursor to the second generation Grubbs catalyst **II** and adjusting temperature and ethylene pressure [44–47]. Most of these studies were performed with model substrates, especially propargylic derivatives such as ethers, esters, thioethers, and included both terminal and internal alkynes. When catalyst **I** was used, a beneficial effect of a heteroatom in propargylic position (especially from an ester or carbonate) in terms of reactivity has been shown, whereas a reverse effect was obtained, when the heteroatom was located in homopropargylic position [41,48]. In the presence of second generation catalysts, unprotected functional groups such as hydroxyl [44] or fluoride [49] were tolerated in propargylic position. EYCM with ethylene has been used in several types of applications in organic synthesis, either to prepare compounds with the final 1,3-diene motive in their structure, or to use them as the first step of a sequential synthesis. The first case is illustrated by the synthesis of Anolignans [48] and another closely related example is shown in the preparation of Amphidinolide E [50–51] where the diene system is extended by further cross-metathesis with 2-methylpenta-1,4-diene (Figure 2).

**Scheme 6:**
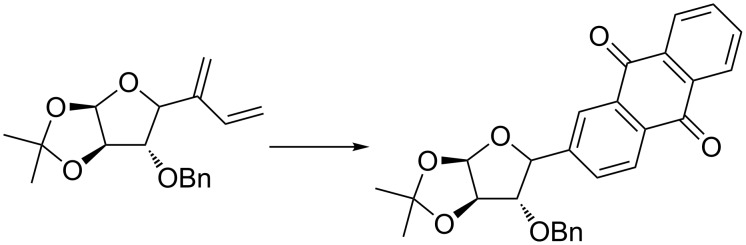
Application of EYCM in sugar chemistry.

The Diels–Alder reaction is one of the most popular transformations of 1,3-dienes. This procedure has been successfully used to prepare *C*-aryl glycoside from *C*-alkynyl glycoside and ethylene according to an EYCM/Diels–Alder/oxidation sequence (Scheme 6) [52–53].

**Scheme 7:**
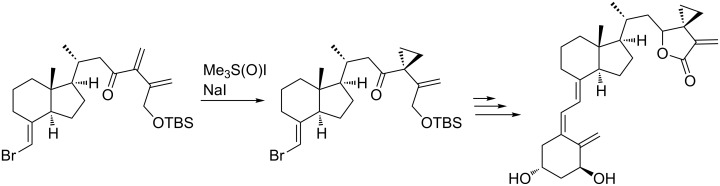
EYCM as determining step to form vinylcyclopropane derivatives.

The selective cyclopropanation of the most electron deficient double bond of the unsymmetrical dienic system has been performed to reach 24,25-ethanovitamine D3 lactones (Scheme 7) [54].

**Scheme 8:**
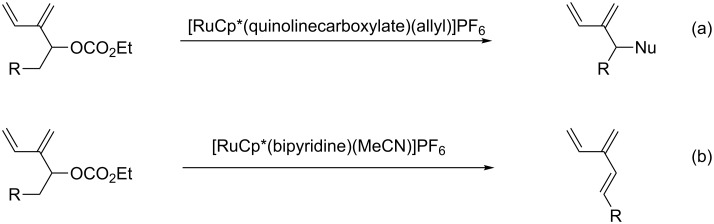
Sequential EYCM with ethylene/nucleophilic substitution or elimination.

Recently, conjugated dienes resulting from EYCM of terminal and symmetrical propargylic carbonates with ethylene have been prepared in the presence of Grubbs second generation catalyst **II**. They have been used in sequential catalytic transformations in the presence of ruthenium catalysts, which are able to perform regioselective allylic substitution by O-, N- and C-nucleophiles (Scheme 8a) [55] and elimination to provide a new access to dendralenes (Scheme 8b) [56].

### Higher olefin–alkyne cross-metathesis

**Scheme 9:**
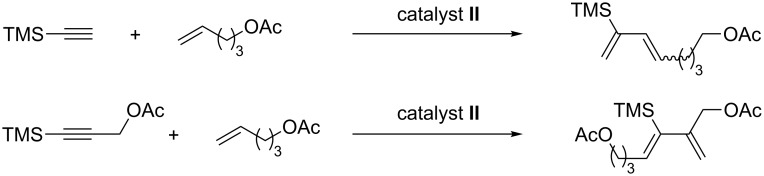
Various regioselectivities in EYCM of silylated alkynes.

This cross-metathesis reaction was introduced in 1997 with the first generation Grubbs catalyst **II** [40] and the initial results indicated that propargylic alcohol derivatives and terminal olefins with oxygen-containing functional groups were well tolerated [57]. As emphasized in the introduction, self-metathesis of the terminal olefin in the presence of a metathesis catalyst competes with EYCM. Essentially for this reason, an excess of olefin with respect to the alkyne (usually from 2 to 9 equiv) was always used to favor complete conversion of the latter. Following the first results and to avoid the competing metathetic reactions, it was shown by Blechert that EYCM reactions could be performed starting from either the olefin or the alkyne substrate bound to a support [58–60]. An improvement of the EYCM was achieved with the second generation Grubbs catalyst **II** starting from terminal alkynes, especially sterically hindered ones. More interestingly, internal alkynes, which were non-reactive with the first generation catalyst, could participate in cross-metathesis with terminal allylic olefins with the second generation Grubbs catalyst [61]. Alkynes substituted by a silylated group have received special attention in EYCM with terminal alkenes in the presence of Grubbs second generation catalyst. It was found that depending on the nature of the alkyne (terminal or internal), the regioselectivity of the cross-coupling changed. Terminal alkynes gave 1,3-dienes with a 1,3-relationship between the alkenyl substituent and the silyl group, whereas a 1,2-relationship was observed starting from internal alkynes (Scheme 9) [62–63]. In addition, the stereoselectivity was low in the first case (3:1) and a single regio- and stereoisomer was obtained from internal alkynes. The regioselectivity was proposed to originate from the steric and stereoelectronic biasing effect of the silyl group during the propagation at the metal alkylidene species. Minimization of steric interactions might be effective during the cycloreversion of the ruthenacyclobutene which is the reason for the observed stereoselectivity. It is noteworthy that internal conjugated diynes protected by a silyl group are also reactive with high regioselectivity [64].

**Scheme 10:**
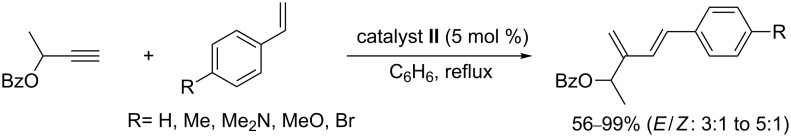
High regio- and stereoselectivities obtained for EYCM with styrenes.

Cross-metathesis of *p*-substituted styrenes with a propargylic benzoate catalyzed by catalyst **II** in refluxing benzene has been performed in almost quantitative yields with perfect regioselectivity leading to 1,3-disubstituted 1,3-dienes and high stereoselectivity in favor of the (*E*)-isomers [65]. The kinetic study of this catalytic system suggests that the formation of an arylidene ruthenium species takes place first; thus, initial interaction of the olefin with the catalyst is preferred (Scheme 10).

**Scheme 11:**
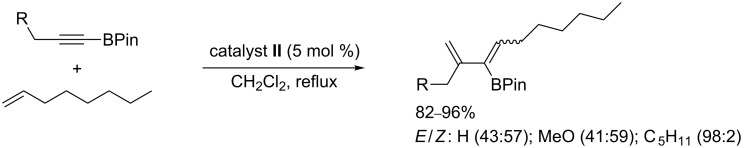
EYCM of terminal olefins with internal borylated alkynes.

Regioselective cross-metathesis was also observed when internal borylated alkynes and terminal alkenes were used as substrates in the presence of catalyst **II** in refluxing CH_2_Cl_2_. The stereoselectivity was found to be very dependent on the substituent both on the alkyne and the alkene (Scheme 11) [66].

**Scheme 12:**
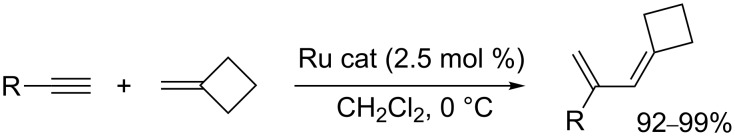
Synthesis of propenylidene cyclobutane via EYCM.

2,2-Disubstituted terminal olefins have scarcely been involved in EYCM. The recent utilization of methylenecyclobutane revealed that second generation catalysts were able to perform the cross-metathesis with a variety of terminal alkynes. The formation of 1,1,3-trisubstituted 1,3-dienes was regioselective and the products were obtained in excellent yields at low temperature (Scheme 12) [67].

**Scheme 13:**
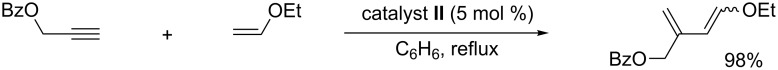
Efficient EYCM with vinyl ethers.

The cross-metathesis of terminal enol ethers with terminal and internal alkynes in the presence of catalyst **II** has led to the regioselective formation of electron rich dienes, precursors of choice for Diels–Alder reactions (Scheme 13) [68]. This is surprising as enol ethers are known to be moderately reactive in olefin metathesis [69] and ethyl vinyl ether is often used to stop ring opening metathesis polymerizations. Vinyl acetate is also a good partner for EYCM under similar conditions.

In the presence of 10 mol % of Grubbs **II** catalyst and 2 equiv of CuSO_4_, the cross-metathesis of terminal alkynes with ethyl vinyl ether led to the expected dienyl ether at 80 °C under microwave heating in toluene, whereas in H_2_O/*t*-BuOH conjugated enals were formed [70].

As already mentioned with dienes resulting from EYCM with ethylene, Diels–Alder reactions have been attempted starting from the more substituted dienes arising from metathesis of alkynes with higher olefins. It must be noted that the regioselectivity of these EYCM always leads to 1,3-dienes with a terminal and a substituted methylene group. It has been shown that the disubstituted double bond possessing a (*Z*)-configuration does not participate in Diels–Alder reactions [61]. Using the EYCM/Diels–Alder sequence, tetrahydropyridines [57], substituted phenylalanines [71–72], modified porphyrins [73], carbocycle-linked oligosaccharides [74] and heterocycles [75–76] were prepared. One-pot EYCM followed by Brønsted acid catalyzed cyclization enabled the formation of monounsaturated cyclic amines [77]. The EYCM of homopropargylic tosylate with allylic alcohol derivatives has been used as a key step for the construction of the side chain of mycothiazole [78].

### Cyclic olefin–alkyne cross-metathesis

**Scheme 14:**
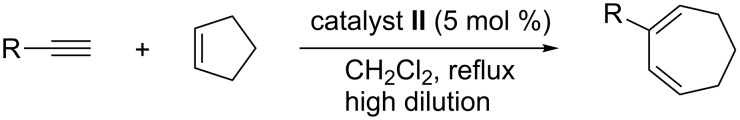
From cyclopentene to cyclohepta-1,3-dienes via cyclic olefin-alkyne cross-metathesis.

As already mentioned, EYCM involving internal linear olefins has not been reported. On the other hand, the cross-metathesis of terminal alkynes with the internal carbon–carbon double bond of cyclopentene has been performed in the presence of catalyst **II** under mild conditions to stereoselectively form expanded 7-membered cycloheptadiene products [79]. To avoid ring opening metathesis polymerization of the cyclic olefin, a special procedure involving high dilution and slow syringe pump addition of the olefin had to be used (Scheme 14). The success of this metathesis reaction demonstrated that ruthenium alkylidene was the active catalytic species (methylidene free conditions).

**Scheme 15:**
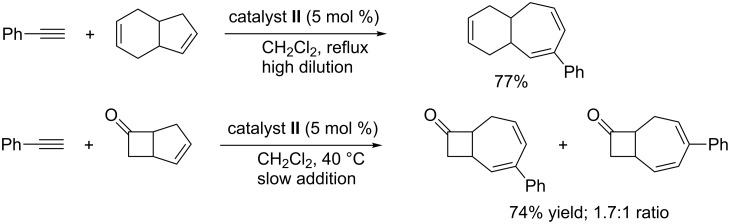
Ring expansion via EYCM from bicyclic olefins.

This ring expansion could be extended to fused cycloalkene substrates such as tetrahydroindene and bicyclo[3.2.0]heptenone, both of them featuring a cyclopentene unit to form functionalized cycloheptadienes (Scheme 15) [80].

**Scheme 16:**
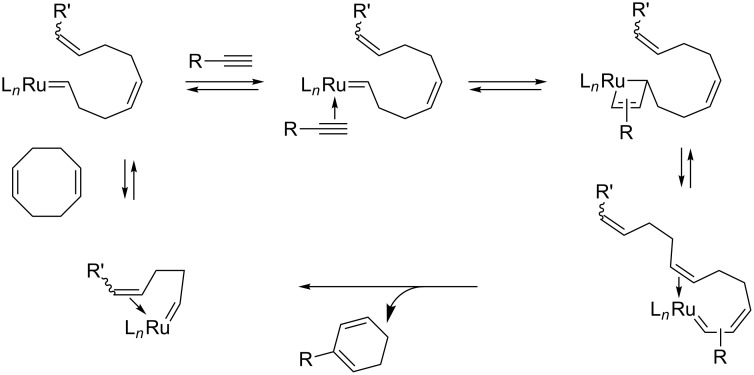
Ring contraction resulting from EYCM of cyclooctadiene.

Starting from 1,5-cyclooctadiene, EYCM also took place with terminal alkynes and the same catalyst, but with this substrate a ring contraction was observed. Conjugated cyclohexa-1,3-dienes were formed in good yields with propargylic and homopropargylic alkynes via methylene-free ene–yne metathesis (Scheme 16) [81]. In these two examples, the driving force seems to be the initial ring opening, which triggers the interaction with the alkyne.

### Diene–alkyne cross-metathesis

**Scheme 17:**
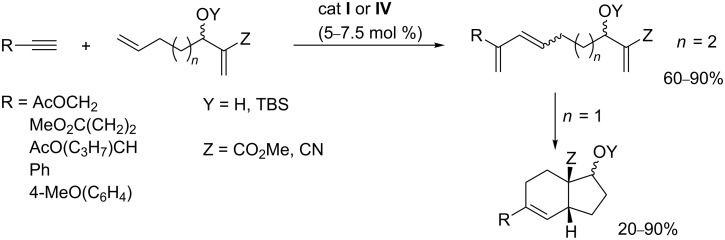
Preparation of bicyclic products via diene-alkyne cross-metathesis.

Very few examples of EYCM of alkynes with non-symmetrical α,ω-dienes have been reported. To perform this type of reaction in a selective manner it is required that the two double bonds have different reactivities with respect to EYCM and that the intramolecular olefin ring closing metathesis is not efficient. Such a reaction has been performed from dienes containing a non-activated double bond and an electron-deficient double bond. Only the non-activated double bond participated in the EYCM. Depending on the chain length in-between the diene functionality and the electron-deficient double bond, the resulting triene could either be isolated or directly cyclized (Scheme 17) [82].

### Ethylene-promoted EYCM

**Scheme 18:**
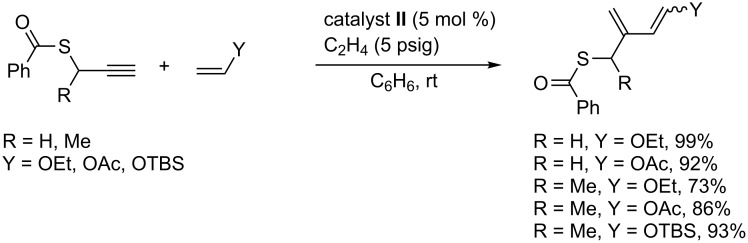
Ethylene helping effect in EYCM.

The positive influence of ethylene in metathesis in the presence of ruthenium catalysts was first evidenced by Mori during the intramolecular ring closing metathesis of enynes [83]. The excess of ethylene would favor the formation of ruthenium methylidene intermediates, and thus prevent catalyst decomposition and maintain catalytic activity. It has also been shown that the presence of ethylene had a beneficial effect either on reactivity or on stereoselectivity in EYCM. The EYCM of vinyl ether, which was successful with some selected alkynes [68], failed when propargylic thiobenzoates were used as alkynes. However, under moderate ethylene pressure (5 psig), the cross-metathesis reaction took place at room temperature (Scheme 18) [84]. The reactivity of various electron rich olefins such as (*tert*-butyldimethylsilyloxy)ethylene and *tert-*butyl vinyl ether was also increased in the presence of ethylene. On the other hand, no improvement of stereoselectivity was obtained under these experimental conditions. It was shown that ethylene increased the lifetime of the Fischer carbene intermediate. Moreover, its role might consist in supporting the methylene transfer, thereby enhancing catalyst turnover.

**Scheme 19:**
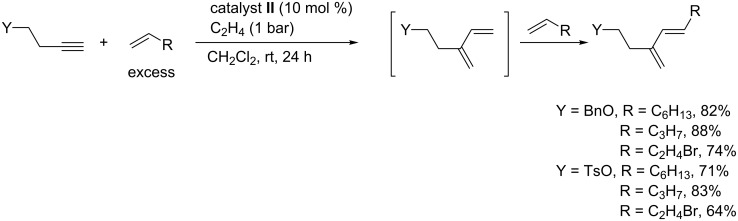
Stereoselective EYCM in the presence of ethylene.

In this process, ethylene was not directly involved in competing ethylene–alkyne cross-metathesis. This was not the case when it was used to perform the cross-metathesis of some homopropargylic alkynes with alkenes that are not functionalized in allylic position. It was assumed that the ethylene–alkyne cross-metathesis producing a conjugated diene was the first catalytic event followed by olefin cross-metathesis of the less substituted double bond of the resulting diene with the alkene partner [85]. The presence of ethylene not only simplified the reaction but also led to a stereoselective cross-metathesis with formation of the (*E*)-isomer as major product (*E*:*Z* ratio > 20:1 and sometimes the (*Z*)*-*isomer was not detected) (Scheme 19). Unfortunately, such high stereoselectivity was not observed when starting from the same alkynes with olefins substituted in allylic position such as 3-(trimethylsilyl)prop-1-ene, 3-*n-*butoxyprop-1-ene and allyl acetate.

### Applications in fatty acid ester derivative transformations

**Scheme 20:**
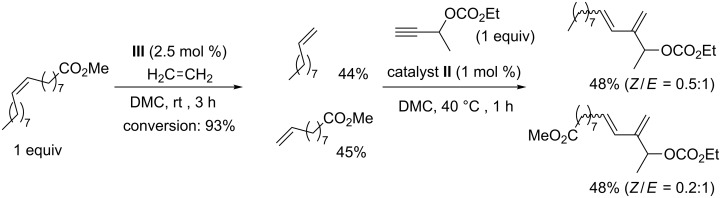
Sequential ethenolysis/EYCM applied to unsaturated fatty acid esters.

The direct transformation of unsaturated fatty acid esters by EYCM has never been performed. This is not surprising as no catalyst has been able to perform the cross-metathesis of alkynes with acyclic internal olefins up to now. This would allow the introduction of a conjugated diene system into an aliphatic hydrocarbon chain. However, the production of terminal olefins upon ethenolysis of unsaturated fatty acid esters followed by cross-metathesis with an alkyne in one pot has recently been carried out in our group using dimethyl carbonate (DMC) as solvent (Scheme 20) [86].

The ethenolysis cleavage was performed in the presence of catalyst **III**, which led to 93% conversion of the starting oleate and selective production of dec-1-ene and methyl undec-9-enoate with trace amounts of octadec-9-ene and dimethyl octadec-9-enedioate resulting from secondary self metathesis of the generated terminal olefins. The cross-metathesis with a terminal propargylic carbonate was then carried out in the same pot with catalyst **II** and led to the formation of dienes. It was not detected that the hindered diene with the carbonate group and the aliphatic chain connected to the same double bond gave a trisubstituted olefin, which indicated a regioselective cross-coupling reaction. On the other hand, the stereoselectivity of the 1,2-disubstituted double bond was not controlled. Under the conditions for the second catalytic step mentioned above, a two-fold excess of olefin was used in a way that only half of the olefin could be transformed by EYCM.

**Scheme 21:**
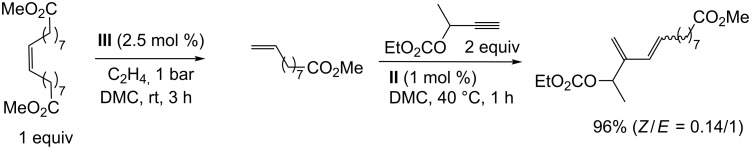
Sequential ethenolysis/EYCM applied to symmetrical unsaturated fatty acid derivatives for the production of a sole product.

The utilization of a symmetrical fatty acid diester arising from self metathesis or selective oxidation of oleate leads to improved experimental conditions as only one terminal olefin was produced upon ethenolysis, and the subsequent EYCM also yielded only one diene (Scheme 21). We have recently shown that a protocol based on syringe pump addition of the alkyne into the reaction medium already containing the olefin and the catalyst precursor, allows to perform the EYCM with stoichiometric amounts of olefin and alkyne. This represents an economical and technical advantage compared to the classical strategy using an excess of olefin [87].

## Conclusion

The EYCM is a very efficient transformation which creates conjugated diene structures with atom economy. It has not been developed as rapidly as the olefin cross-metathesis because it suffers from some reactivity and selectivity issues that have not yet been solved. Regioselectivity of the cross-coupling reaction is usually good as no problem is encountered starting from symmetrical alkynes; and in the case of unsymmetrical alkynes, the products featuring less steric hindrance are formed. The stereoselectivity of the newly formed trisubstituted double bonds is still not controlled. It must be noted that no EYCM starting from acetylene has been described. On the contrary, polymerization of acetylene in the presence of ruthenium carbene complexes has been reported [11]. A challenge that has still to be faced is the EYCM starting from acyclic internal olefins.
